# Undergraduate medical education in the U.S. and Israel: contrasts and common challenges

**DOI:** 10.1186/s13584-015-0053-4

**Published:** 2015-11-12

**Authors:** Arthur M. Feldman

**Affiliations:** Temple University School of Medicine, 3500 N. Broad Street, Suite 1150, Philadelphia, 19107 PA USA

## Abstract

In 2014, the Israeli Council for Higher Education (CHE) commissioned an international panel of outstanding educators to prepare an *ad hoc* report reviewing the four established medical schools in Israel. The report described the strengths, weaknesses and challenges facing medical education in Israel with a focus on three specific areas: workforce planning, the structure of the curriculum and the financing of medical education.

There are interesting parallels between the challenges facing medical education in the U.S. and in Israel: a lack of clarity regarding the optimal size for the workforce and the optimal method for enhancing the number of primary care physicians; an absence of methodologies for evaluating innovations in medical education and a lack of transparency in funds flow. However, there are also important differences, one of the most important being an absence in Israel of students’ hands-on responsibility for their patients until year six of their undergraduate medical education.

The presence of a small number of medical schools with common funding and geographic proximity, in a relative sense, provides the Israeli medical schools with a unique opportunity to evaluate innovations in medical education and to set a high bar for inter-school collaboration and cooperation.

## Background

In 2014 the Israeli Council for Higher Education (CHE) commissioned an *ad hoc* committee composed of internationally respected physician-educators (four from the U.S., two from the U.K. and two from Israel) to provide an external review of Israel’s four established and accredited medical schools. In a recent IJHPR article, the report’s authors discuss three inter-related topics that bridge medical education and health care delivery: planning the physician and healthcare workforce to meet the needs of Israel’s population in the 21^st^ century; enhancing the coordination and efficiency of medical education across the continuum of education and training; and the financing of medical education. The members of the committee undertook an enormous task and have provided a comprehensive and scholarly assessment of the strengths and weaknesses of undergraduate medical education (UME) in Israel as well as the challenges faced in educating the next generation of physicians. Many of these challenges cross borders and continents; the overwhelming amount of new knowledge emanating from research laboratories and clinical trials, the financial stresses on hospitals and physicians that limit the resources that can be allocated to education, and pressures to shorten hospital stays and increase ambulatory care. How individual medical centers face those challenges is predicated in part on the influence of regulatory agencies in each country, the support – or lack of it – from universities, governments, and philanthropies, and the external influences of local and national politics [1]. However, the CHE report provides an opportunity to compare and contrast how Israel and the U.S. have approached the fundamental issues that face each of us and to explore ways in which lessons learned in the U.S. might inform efforts to reform UME in Israel.

### Workforce planning

There are both interesting similarities and significant differences in “workforce planning” between Israel and the U.S. – although in both countries the term “workforce planning” might be described as an oxymoron. The CHE Report points out correctly that workforce planning in the U.S. is “highly fragmented” and experts cannot agree whether there is an actual physician shortage or whether the problem can be solved by better geographic distribution and an increase in the percentage of medical school graduates who pursue careers in primary care [2]. The latter view fails to recognize that an aging population requires not only primary care physicians but subspecialists who have expertise in caring for patients that have diseases that are over-represented in an aged population: degenerative joint disease, age-related macular degeneration, coronary artery disease, heart failure, critical care medicine, neurodegenerative diseases and cancer [3, 4].

The paucity of U.S. students who pursue careers in primary care has been attributable to the high medical school debt of U.S. students - ~ $180,000 [5]. The fact that Israel has a similar shortage of primary care physicians despite substantial governmental support and far lower tuition costs suggests that career decisions are made on factors that are more complex than financial exigencies alone [6]. Both the CHE task force and U.S. primary care groups have proposed innovative programs to increase students’ interest in primary care: early immersion in a primary care setting, identification of ideal role models, and enhanced compensation models [7]. Unfortunately, none of these efforts have effectively shifted students’ interests. The current process of expanding Israel’s medical schools will help mitigate the existing and expected shortages in primary care physicians, but apparently this will not suffice and additional solutions are needed. Dr. Schoenbaum and his colleagues have proposed in the CHE report a concept that is gaining substantial interest in the U.S. for mitigating the shortage in primary care physicians: an increased use of non-physician clinicians as primary care providers [8]. While there is presently a limited number of non-physician clinicians in Israel, the opportunity to train physician assistants in a medical school environment using the model first described by Dr. Eugene Stead at Duke a half-century ago may mitigate some of the workforce issues while at the same time providing a new opportunity to increase medical school revenues.

The CHE report notes that the U.S. medical workforce is less dependent on foreign trained physicians than the Israeli workforce. This should not be construed as being a disadvantage for Israeli medicine because the demographics of the foreign trained workforce in the two countries are quite different. The majority of foreign-trained physicians who enter the Israeli workforce are Israeli citizens. By contrast, the majority of foreign-trained physicians who enter the U.S. workforce are foreign nationals who graduate from nearly 2,000 different medical schools worldwide including many from countries with whom the U.S. has relationships that are at times problematic. Without information about their schools of origin, these post-graduate trainees are selected based almost exclusively on scores on the USMLE examination; a test that provides limited information about a physician’s clinical capabilities. As a result there is wide variation in how these physicians perform on subsequent testing for licensure in the U.S. [9, 10]. However, those who become licensed in the U.S. appear to perform well in practice although acculturation is an important but not obligatory part of their training. U.S. citizens who enter the U.S. medical workforce from abroad are most commonly graduates of for-profit medical schools in the Caribbean Islands that are unregulated, often graduate over 1,000 students per year, and farm their students out to U.S. hospitals for clinical clerkships by compensating the hospitals at a rate exceeding $500 per student per week – a reimbursement strategy that is problematic for a U.S. medical school [11].

### Enhancing the coordination and efficiency of medical education across the continuum of physician education and training

#### The structure of pre-clinical and clinical UME

The construct of UME in Israel differs substantively from that in the U.S. The most important difference between UME in the U.S. and Israel appears to be that Israeli students have less substantive contact with patients. William Osler, the father of American medical education, pointed out a century ago that: “To study the phenomenon of disease without books is to sail an uncharted sea, while to study books without patients is not to go to sea at all. [12]. Students are benefited when they are exposed to patients from day one of medical school and by incorporating clinical cases into basic science instruction or problem based learning [13]. During the clinical years students should be an integral part of the medical team – caring for assigned patients under the guidance of a resident and a faculty member rather than simply observers, and they should not be allowed to have “jobs” or any other extra-curricular activities that limit their participation in the medical curriculum and in particular, their clinical rotations. Not mentioned in the Report is the valuable role that standardized patients have played in the U.S. for nearly two decades [14]. They help students learn how to interview and examine a patient, they allow students to hone their clinical skills, and they provide both summative and formative feedback for the students. More recently, new technologies have supplemented the traditional standardized patient format in order to optimize assessment and evaluation such as adding Google glasses [15]. Small group workshops that use role play prior to patient interviews as well as videotaping patient encounters followed by group discussion have also proved useful [16]. In the U.S., a relatively new standard for medical education is inclusion in the curriculum of “translational medicine” [17]. Some schools have approached this by including instruction in the social sciences, statistical analysis, population health, the fundamentals of healthcare safety and quality, bioethics and health care finance. We believe that wherever possible students should have hands-on experience in translational research; however, this is not practical for schools that do not have programs in translational research. This could be an opportunity to link medical education in Israel with the robust and medically oriented Israeli biotechnology industry and the outstanding basic and translational science laboratories at Israeli academic institutions.

Dr. Thomas Nasca, the President of the American College of Graduate Medical Education (ACGME),m pointed out that medical school graduates today are an “undifferentiated primordial mass that must be shaped during residency” into medical practitioners [18]. Thus it is critical that UME and GME be viewed as a continuum during which a student progresses from a student to a practicing physician as they develop increasing competencies [19]. Each pre-clinical block and clinical clerkship should have well defined core competencies that each student is expected to attain. As individual specialty and subspecialty organizations develop milestones and entrustable professional activities (EPA’s) for GME, these concepts should be introduced into the undergraduate curriculum and incorporated into the assessment and evaluation of each student [20]. The CHE Report suggests that grading students based on achieved milestones might provide an opportunity for students to transition from a student to a resident based on their individual timeframe for understanding and using information. While intriguing, the complexities associated with assessing, tracking and scheduling hundreds of students based on individual metrics would likely be problematic if for no other reason than the need for additional support staff and the attendant costs.

#### Innovation

The authors of the CHE Report were clearly influenced by the 2010 Carnegie Report on UME and the recent survey by Nara et al. [21, 22]. These reports posited that there is a need for medical schools to decide whether to “continue in the directions established over a hundred years ago [by the 1910 Flexner Report] or take a fundamentally different course guided by contemporary innovation and new understanding about how people learn”[21]. It must be remembered that the definition of “innovation” is a “new idea or method” but not one that has necessarily proven successful. Thus, while changes such as flipped classrooms, active learning, e-learning, team learning, and simulation have been implemented at some – but certainly not all – U.S. medical schools, they have not yet undergone a thorough evaluation because the requisite tools have not been developed and it often takes many years to reach measurable endpoints [23]. In addition, many of the innovations in UME are expensive. For example, flipped classrooms and active learning require the availability of a large teaching faculty to facilitate small group discussions, multiple small classrooms and a collection of professionally produced on-line lectures. These novel teaching tools can be incorporated into the curriculum in selected areas rather than across the entire curriculum. For example, in our own curriculum, we use the more traditional lecture format for didactics in anatomy, biochemistry, immunology and microbiology. The remainder of the curriculum is system-based with approximately 40 % of the curriculum presented in the lecture format.

The CHE Report raised the concern that there were limited sites for clinical clerkships in Israel due to a paucity of hospital beds per capita and an over-reliance on hospital versus ambulatory teaching. This raises two possibilities. First, the possibility of increasing the number of clerkship opportunities for students by decreasing the number of patients on each clinical team could be evaluated. Kenneth Ludmerer pointed out in his book *Let Me Heal* that in an era of cost containment, a resident’s primary job is to churn patients through the hospital system as rapidly as possible [24]. This obviates the ability of students and residents to see the full spectrum of a patient’s disease, to have the time to get to know each patient and to ensure that students have appreciated the key elements of the history and physical examination. Students are invariably part of large teams. Two innovative programs have taken a very different approach. Hopkins Bayview (The Aliki Initiative) and the Brigham and Women’s Hospital have created teams with half the normal complement of patients so that students and residents have more time to spend with each patient [25, 26]. The Aliki Initiative is associated with higher patient satisfaction, higher resident satisfaction and improved patient outcomes although the cost of restructuring the clinical teams might be prohibitive. Second, opportunities for increased outpatient exposure should be evaluated. In the U.S., there is a strong financial incentive for providing as much care as possible in the outpatient environment. In fact, many health care economists and policy pundits posit that the hospital of the future will be a large intensive care unit – much of today’s care being administered in outpatient clinics. How to compensate busy outpatient physicians for teaching will be the challenge of increasing outpatient exposure albeit with a strong societal benefit.

### Financing of medical education

Medical education financing is a universal problem with enormous differences in UME and GME funds flow across different countries and in the U.S., across different medical schools: a problem that has increased as reimbursements have precluded cost-shifting from practice plans and hospital revenues to support the academic missions of the medical school [27]. At research-intensive medical schools in the U.S. tuition does not begin to cover the medical school budget because Federal funding does not cover the cost of research even if all investigators were optimally funded. Research-intensive medical schools in the U.S. therefore depend on university support, endowments, philanthropy and commercialization of intellectual property to support the research enterprise and in some cases, obtain support from associated health systems. An increasing number of medical schools in the U.S. have received significant financial contributions from wealthy individuals whose names are now incorporated into the name of the medical school: for example, Weill Cornell Medical College, Icahn School of Medicine at Mount Sinai, and Warren Alpert Medical School at Brown University. By contrast with Israeli schools, many U.S. medical schools compensate basic science teachers based on the number of hours they teach and the number of hours required to prepare a lecture. As a result, tenured faculty who no longer have grants to support their salaries often increase the time they spend teaching. For clinical faculty who spend significant amounts of time with students on their inpatient or outpatient services, many U.S. medical schools lower the relative value unit (RVU) expectations commensurate with the time they teach or pay teachers directly for their services [28]. This is important because without incentives, both pre-clinical and clinical educators are less likely to teach. Many medical schools have also developed multiple pathways to promotion in order that educators can be promoted based on their teaching evaluations and education scholarship rather than on extramural funding and the number of scientific publications and in some institutions educators can also achieve tenure. These incentives are critical for not only attracting teachers but also in making them feel that they are an important component of the medical school and university.

Lost in discussions of U.S. medical school financing is the fact that medical schools that do not or cannot support a research program have an economic profile that is significantly better than that of the traditional research-intensive schools [17]. In fact, a publicly traded company owns at least one offshore for profit medical school. In addition, the Liaison Committee on Medical Education (LCME) recently accredited the first for-profit allopathic medical school in the U.S. and for-profit osteopathic medical schools have previously been approved in the U.S. We have argued that it is important for medical students to have exposure to physician-scientists and clinician investigators who pursue translational research in either laboratory or clinical research centers while at the same time caring for patients [29]. Exposure to these research-oriented clinicians provides important role models, the opportunity to have hands-on participation in research activities and a perspective on medicine and medical education than is different from that which a student would obtain when their instruction is in a community hospital. The absence of physician-scientists and clinician-investigators at new medical schools and at for-profit medical schools threatens to create a two-tiered system of medical education in the U.S., yet without resources, more and more U.S. medical schools may shift to the second tier model. Thus, the presence of governmental funding for medical education in Israel is a critically important financial foundation that should be continued and increased as necessary.

### Unique opportunities for Israeli medical education

Israel has only five medical schools – the four established schools covered in the report and a new medical school in the Galilee. The existence of only five medical schools which are largely funded by the government through the Council for Higher Education and which are located relatively close to one another, provides unique opportunities for both UME and GME. First, as pointed out in the CHE Report, there is the opportunity to share resources. The best lecturers and the best lectures from across the five schools can be used for the didactic portion of pre-clinical courses across the schools. In an era when many medical schools are implementing innovative but unproven and often expensive new teaching formats, there is also an opportunity to use the five Israeli medical schools as innovation incubators to actually test whether one educational strategy is better than another. Outcome metrics for comparisons of different education formats can include scores on standardized tests, OSCI’s or even oral examinations. The outcome of these studies would be useful to Israel for optimizing the educational experience for students and student outcomes as well as benefiting medical schools in other industrialized countries.

The economies of scale across the five schools might also provide novel opportunities for the medical schools to create ambulatory care facilities that could provide a collaborative learning environment for students from multiple schools. If these facilities are built in areas that are underserved in medicine, the creation of joint programs focused on health disparities and population health could also decrease health disparities that have occurred in ethnic or economically deprived areas. The five medical schools should also work together to lobby the government to transition from a university-centric funding model to a medical school-centric funding model as there is little rationale for the funds flow coming through the university as it is unlikely that this can be done with complete transparency. Furthermore, there will always be questions as to whether the universities apportion some of the funds for university infrastructure rather than for direct support to UME. The five medical schools should also look for opportunities to participate and actually lead the development of new physician assistant and nurse practitioner programs. By embedding these programs in the medical school both medical students and non-physician clinicians are exposed to the concepts of inter-professional education and learn to work seamlessly as a part of the care delivery team.

Israeli medical schools should also take advantage of the enormous investment in biotechnology in Israel by creating medical school-based incubator facilities and early stage venture funds to take advantage of intellectual property coming out of medical school laboratories. This effort should be collaborative across the five medical schools because the expertise of each may be distinct yet synergistic - making the aggregate intellectual property more valuable when bundled rather than put out to the market as individual pieces. Finally, Israeli medical schools should follow the lead of successful U.S. medical schools in pursuing philanthropy from both Israel and the U.S. that supports all three missions of the academic medical center: teaching, research and patient care. The message to potential donors and to grateful patients is the same in the U.S. as in Israel – without a continuing supply of new medical graduates with the skills to provide effective and efficient care in the short term and the ability to be learners throughout their careers, the health of the nation will suffer.

## Conclusions

There are both differences and similarities between medical education in the U.S. and Israel. Important differences could be mitigated by focusing restructuring on: increasing the clinical exposure for medical students both during the pre-clinical and clinical years of medical school; eliminating student “moonlighting;” evaluating innovative methods for restructuring clinical teams in order to provide more clerkship opportunities; enhancing utilization of outpatient clinics for student education; developing clear rewards for educators including new pathways to promotion and tenure; establishing transparent funds flow for both pre-clinical and clinical educators; and financing undergraduate medical education through direct funds flow to medical schools rather than through university finance offices. Finally, the presence of only five medical schools within a relatively small geographic footprint should provide an opportunity for the schools to collaborate and cooperate in medical education in order to lower costs, optimize patient and space resources and take advantage of the unique academic and scientific strengths of the medical schools in Israel.
